# Bilateral Endoscopic Medial Maxillectomy for Bilateral Inverted Papilloma

**DOI:** 10.1155/2012/215847

**Published:** 2012-07-02

**Authors:** Satoru Kodama, Toshiaki Kawano, Masashi Suzuki

**Affiliations:** Department of Otolaryngology, Faculty of Medicine, Oita University, 1-1 Idaigaoka, Hazama-Machi, Yufu, Oita 879-5593, Japan

## Abstract

Inverted papilloma (IP) is a benign tumor of the nasal cavity and paranasal sinuses that is unilateral in most cases. Bilateral IP, involving both sides of the nasal cavity and sinuses, is extremely rare. This paper describes a large IP that filled in both sides of the nasal cavity and sinuses, mimicking association with malignancy. The tumor was successfully treated by bilateral endoscopic medial maxillectomy (EMM). The patient is without evidence of the disease 24 months after surgery. If preoperative diagnosis does not confirm the association with malignancy in IP, endoscopic sinus surgery (ESS) should be selected, and ESS, including EMM, is a good first choice of the treatment for IP.

## 1. Introduction

Inverted papilloma (IP) is a common benign tumor of the nasal cavity and paranasal sinuses. IP is confined to one sides of the nasal cavity in most cases, and bilateral IP is extremely rare with only a few cases reported in the English literature [1, 2]. Herein, we describe a large IP that involved both side of the nasal cavity and extended into the orbita, mimicking association with malignancy. The tumor was successfully treated by bilateral endoscopic medial maxillectomy (EMM).

## 2. Case Presentation

**Figure 1:**
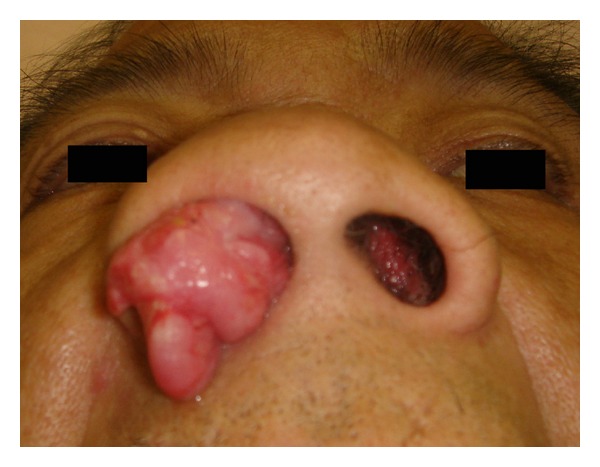
Preoperative view of the nose. The huge tumor filled in the bilateral nasal cavity. The tumor spread from the right to the left.

**Figure 2:**
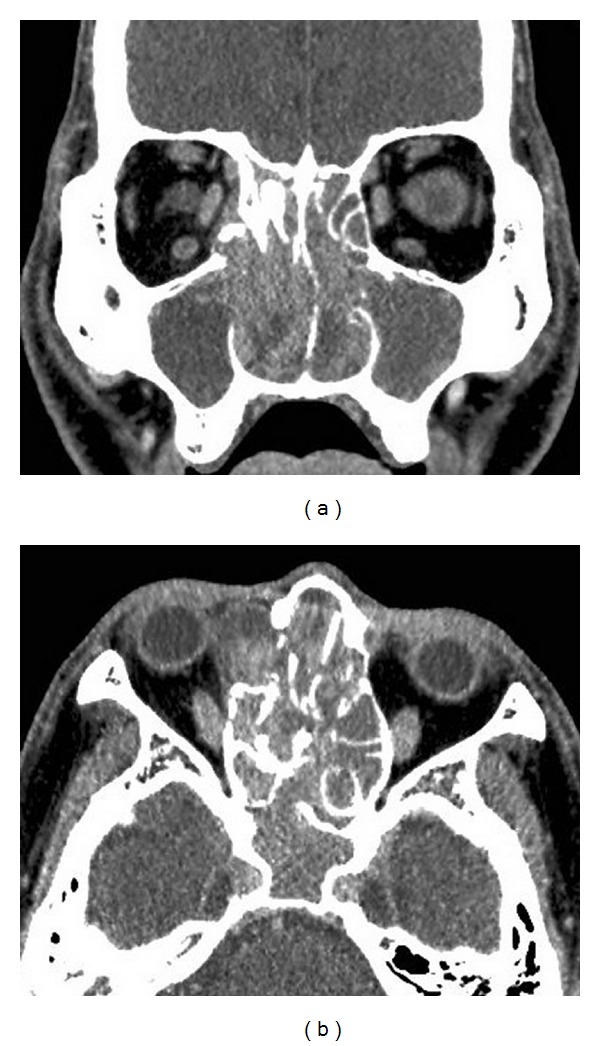
Enhanced computed tomography (CT) image (a, b). A homogeneously enhancing mass that filled the bilateral nasal cavities and the maxillary and ethmoid sinuses. Bone destruction of the right orbita was observed. Osteogenesis was observed in the right ethmoid sinus.

A 55-year-old man presented with bilateral nasal tumor that caused progressive bilateral nasal obstruction. He could not breathe through the nose. Anterior rhinoscopy revealed a pinkish mass that filled both nasal cavities (Figure 1). The tumor could be also observed orally, in the pharynx. Enhanced computed tomography (CT) showed a homogeneously enhancing mass that filled the bilateral nasal cavities and maxillary, ethmoid, and sphenoid sinuses. The tumor extended into the right orbita causing bone destruction. No bone damage was observed at the skull base. Osteogenesis was observed in the right ethmoid sinus (Figure 2). The frontal sinuses were poorly pneumatized. The base of the tumor was considered to be at the right ethmoid sinus. Biopsy revealed IP; however, association with malignancy could not be ruled out because of local aggressiveness. No neurological or visual defects were observed. Endoscopic sinus surgery (ESS) was then performed under general anesthesia. The anterior portion of the tumor, protruding from the nose, was cut using the harmonic scalpel (HS). The tumor was removed piece by piece with the HS to avoid bleeding. Intraoperative frozen section examinations were repeated suspecting a malignancy; however, the results showed all IP without malignancy. Since the tumor was widely spread in the maxillary sinus in both sides, the medial maxillary wall was incised, and EMM was performed in both sides. Enlarged resection of the lateral wall allowed complete removal of the tumor in the sinuses. The left nasolacrimal duct was preserved; however, the right lacrimal sac and duct were cut and removed due to the tumor involvement. The base of the tumor was identified at the roof of the right ethmoid sinus with a neogenerated bone and was removed along with surrounding mucosa.The bone of the skull base was preserved.The tumor in the orbita was also removed completely, and the periosteum of orbita was left intact. Intraoperatively, a septal perforation due to septal invasion of the IP was observed, and the tumor on the right side penetrated in to the left side through the perforation at the posterior part of the nasal septum. Thus, the tumor was an advanced unilateral tumor that has eroded into the other side by erosion of the septum. In the progressive dissection, the tumor was completely removed in both sides, and the surgical margin was free of disease. The operating time was 5 hours, and intraoperative blood loss was 300 mL. Permanent histopathologic examination revealed IP without malignancy. The postoperative course was uneventful, and no recurrence was observed at the 2-year follow-up examination.

## 3. Discussion

IP is a benign tumor of the nasal cavity and paranasal sinuses. The management of sinonasal IP is often complicated due to its local aggressiveness, high recurrence rate, and association with malignancy. The accepted treatment of choice is complete wide local resection. Several external surgical procedures, such as lateral rhinotomy, medial maxillectomy, and the midfacial degloving approach, have been performed for extended IPs [1, 3]. ESS is now widely accepted and commonly performed in cases requiring nasal or paranasal sinus surgery. ESS provides superior magnification, illumination, and angled visualization, thereby allowing the surgeon to isolate the base of the tumor and accurately define the extent of disease. The assessment of endoscopic resectability depends on preoperative endoscopic findings and imaging. The skill of the surgeon is also an important factor when selecting a surgical technique. Surgeons with extensive training and experience in ESS would likely be more comfortable in utilizing endoscopic techniques as the extent of the disease increases. Recently, EMM has been validated as an effective treatment for IP [3, 4]. Removing the medial wall of the maxillary sinus by using an endoscopic approach results in a recurrence rate comparable to that of traditional open medial maxillectomy. In the present case, although the tumor was an advanced unilateral tumor that has eroded into the other side by erosion of the septum and filled in bilateral maxillary sinus, EMM was effective for the complete removal of IP with a good visualization and a wide working space. The advantages of EMM are lack of facial or gingival incision, lack of external scar, no loss of bony nasal or anterior maxillary support, low risk of infraorbital nerve paresthesia, shorter hospital stay, less postoperative pain and facial swelling, less blood loss and crusting, and low morbidity [5]. Bilateral IP is extremely rare; to our knowledge, the case reported here is the first that included resection by bilateral EMM that was completed without complications. 

 In conclusion, ESS could be successfully used to achieve the complete removal of a large tumor such as an IP that affects bilateral nasal cavities and sinuses. If preoperative diagnosis does not confirm the association with malignancy, ESS should be selected for IP. ESS, including EMM, is a good first choice of the treatment for IP.
